# Students’ perception of problem-based learning at a Japanese medical school: an exploratory sequential mixed method

**DOI:** 10.5116/ijme.6399.dee1

**Published:** 2022-12-26

**Authors:** Htain Lin-Aung, Daisuke Masumoto, Zayar Linn, Yusuke Kobayakawa, Satoshi Okamura, Kosuke Kurihara, Kunimasa Morio, Yasura Tashiro, Hiroyuki Sakurai, Hiroki Hori

**Affiliations:** 1 Department of Medical Education, Mie University Graduate School of Medicine, Tsu, Japan; 2 Department of Pediatrics, Daido Hospital, Nagoya, Japan

**Keywords:** Problem-based learning, student’s perception, Japanese medical students

## Abstract

**Objectives:**

This study aimed to assess the perception of PBL among Japanese medical students.

**Methods:**

Learning effects and challenges of PBL from the students’ viewpoint were assessed with an exploratory sequential mixed method. Focus group discussions followed by thematic analysis were conducted with 27 students and residents. Then a questionnaire survey was carried out. A total of 119 out of 258 students (46.1%) responded. The results from 24 questions were analyzed with a residual analysis.

**Results:**

Thematic analysis extracted 14 themes from four discussion topics. The participants in focus group discussion regarded the PBL program as a better learning method than lectures. But some key phrases on the challenge of social interaction, including reluctance to actively discuss and collaborate with unfamiliar peers, were found. The questionnaire survey revealed a significantly lower adjusted standardized residual (ASR) for the positive response in five of six questions in the category of social interaction; improvement of communication skills (ASR = -3.303, n = 118, p < .001), enhancement of responsibility at group discussions (ASR = -2.078, n = 119, p = .038), building social networking (ASR = -3.006, n = 119, p = .003), becoming to sympathize with patients (ASR = -2.449, n = 119, p = .014) and understanding social aspects of clinical practice (ASR = -5.790, n = 119, p < .001). Conclusion: The Japanese medical students perceived PBL as an effective learning strategy. However, they had a problem with social interactions.

## Introduction

After the introduction of problem-based learning (PBL) into medical education at McMaster University in Canada in the 1960s,^1^ PBL has been applied to medical education curricula around the world. Previous studies reported that PBL is advantageous for knowledge retention, attaining problem-solving abilities, and integration of basic science and clinical medicine.^2^^,^^3^ Moreover, PBL is assumed to enhance social learning and promote interpersonal communication and presentation skills.^4^^-^^6^ In addition, it was reported that PBL encourages self-directed learning and increases student’s enthusiasm and motivation for learning.^7^ On the other hand, factors affecting the effectiveness of PBL programs have been reported.^8^^,^^9^ These include the quality of case scenarios at tutorials, tutor’s tutoring ability, active participation of student-peers, and the process of self-learning.

Mie University Faculty of Medicine in Japan introduced the concept of PBL into the 6-year medical education curriculum in 1995. The original program was planned as PBL tutorials for the 3rd to 4th year students in pre-clinical education. However, it faced some problems such as student’s motivation for self-learning and a shortage of tutors. The staffing issue became more serious in association with the increasing number of students. Furthermore, some faculty members and students complained about the replacement of conventional lectures with PBL tutorials. To solve these issues, the pre-clinical education program was amended to operate with limited resources. In addition, it aimed to match the preference of the faculties for lectures although it may be criticized for deviating from authentic PBL tutorials. The program was modified to be composed of self-learning, PBL tutorials, team-based learning (TBL), and lectures. The modified program has continued for more than 10 years. However, the program may have weaknesses in facilitating active learning of the students such as tutors feeling that students have a passive attitude at PBL tutorials. A previous study conducted during the introductory period of PBL in Asia reported the incompatibility of Asian cultural attitudes with active learning methods.^10^ The study reported characteristics of Asian students, such as disliking open criticism and lacking motivation to ask questions, that made the use of such methods difficult. A recent article from Japan reported that some Japanese medical students may be reluctant to shift towards active learning because they have become accustomed to passive learning since childhood.^11^

To clarify learning effects and challenges of a PBL program conducted in parallel with lectures for Japanese medical students, we studied students’ perception of the program. Because there are few reports focusing on this issue, we adopted an exploratory sequential mixed method for the analysis. This methodology provides a broader spectrum of ways to understand students’ perception in different contexts than those that could be done through either quantitative or qualitative approaches alone.^12^ Our findings may contribute to the improvement of pre-clinical education at medical schools with similar problems in Japan and other Asian countries.

## Methods

### Medical education curriculum

At Mie university, medical students join liberal arts education and community-based education in their 1st and 2nd years. In these years, they also learn basic medical sciences by attending lectures. In the 2nd and 3rd years, they participate in practical trainings in basic sciences. Afterwards, students move to a preclinical education curriculum composed of PBL tutorials, TBL, lectures, clinical skill training, and laboratory internship in the 3rd and 4th years. Then the students participated in clinical clerkship for 70 weeks.

### PBL program

In the PBL program, a total of 125 students were allocated into 16 groups. Each group consisted of seven to eight students who took part in collaborative learning. They were requested to practice self-directed learning before and after group work. The PBL program contains 10 units, which were organized according to organ systems. A single unit was carried out for 4 to 6 weeks. The units were organized into three types of group studies: PBL tutorials (Units 2, 3, 6, and 8), mixture of PBL tutorials and TBL (Units 1, 4, 5, and 7), and exclusively TBL (Units 9 and 10). In addition, lectures related to the topics of the PBL programs were given. A PBL tutorial session was facilitated by a tutor for each group. A TBL session was carried out without the tutor facilitation. Instead, one to two teachers guided a whole-class activity in a single classroom, and students dealt with tasks at a single table with members of their group. The formal PBL tutorial had two sessions per week for analyzing and reporting. Each session time was set to 90 minutes. Students were sequentially provided with a patient scenario containing information on medical interview, physical examination, laboratory tests, and therapeutic intervention. Through discussions at each tutorial, students were expected to improve skills of problem distillation, problem-solving and clinical reasoning. Students were evaluated with a portfolio submitted in every group session, a paper test, student-and-peer assessment, and tutor's assessment at the end of each unit. The peer and tutor assessments were carried out in three domains of learning: self-learning in preparation, active participation in group discussions, and cooperative attitude in group work. A tutor attending the tutorials evaluated all students in the group. The tutors also provided formative feedback to the students. TBL consisted of one session of 120 minutes a week. The supervising teacher gave the students learning topics, and then they had a group discussion on the topic. After the discussion, several groups presented a summary of their discussions at a plenary session, which was followed by a small lecture by the supervising teacher. Students were evaluated for their learning achievements similarly to the PBL tutorials, but a tutor assessment was not included in TBL.

### Study design

This study employed an exploratory sequential mixed method to understand students’ perception systematically by integrating qualitative and quantitative findings. In the first phase, qualitative data collection and analysis were performed using focus group discussions (FGDs) and a thematic analysis. Based on the results, we explored students’ perception of PBL and identified specific themes. Then we developed a questionnaire and conducted a questionnaire survey in the second phase to measure the students’ perception quantitatively. This process corresponded to “the building approach” of the exploratory sequential mixed method. Furthermore, “the merging approach” was applied through integration and visualization of the findings at each phase.^13^^,^^14^

FGDs were performed in 2019. FGDs were held in four groups comprising of students or residents who joined the PBL program in the same academic year. All participants were Japanese. The participants were requested to discuss their experiences of the PBL program at Mie University Faculty of Medicine. The research questions at the first phase were: 1) outcome of PBL, 2) application of PBL outcome, 3) challenges of PBL, and 4) suggestions for better PBL. The actual questions to the participants were as follows: 1) What ability and knowledge have you obtained from the PBL program? 2) How would you apply the ability and knowledge that you obtained through the PBL program to your later academic life? 3) What challenges and difficulties of the PBL program did you face? 4) What advice would you give to make the PBL programs better? The group discussion was facilitated by an investigator who was not involved in the management of the PBL program. The discussion lasted about 90 minutes for each group. The discussion was recorded using a voice recorder and transcribed verbatim into written documents. The data were anonymized throughout the process. The discussions were carried out in Japanese. The textual documents were translated from Japanese to English, reviewed by three investigators and then analyzed. Thematic analysis was conducted based on the six-phase framework by Baum and Clarke.^15^^,^^16^ The words and phrases used in the discussion were coded with Atlas.ti MacOS Software Version 8.3. Two investigators reviewed the result and defined the themes.

To quantitatively assess the students’ perception of PBL, a cross-sectional questionnaire survey was conducted among 5th and 6th year students in 2019. The research questions at the second phase were developed based on the results of the thematic analysis. A self-administered anonymous questionnaire was distributed by hand and by e-mail if requested. The answered questionnaire was posted in a collection box in the classroom. We analyzed responses posted within two months from distribution. A questionnaire constructed using the results of the qualitative study was used in the survey. The questionnaire was composed of several categories defined according to the research questions at the second phase. The collected data were analyzed for each question and category. The information of student characteristics was also collected with the survey, which included sex, age, school year, and the result of the computer-based test (CBT). CBT was carried out just after the PBL program by the Common Achievement Tests Organization in Japan for the qualification of entering clinical clerkship. Students self-reported their rank of CBT among their classmates. The questionnaire was produced in English at first, translated into Japanese, and validated by counter-translation into English before the survey. Students responded to 5-point Likert scale questions. The definitions of each point in the scale were as follows: 1, strongly disagree; 2, disagree; 3, neither agree nor disagree; 4, agree; and 5, strongly agree. For a question item with negative meaning, reversed scoring was adopted for data analysis. The responses to each question in the questionnaire survey were divided into three classifications: strongly agree/agree, neutral, or strongly disagree/disagree in the analytical process. The result was tested for independence with a contingency table, and then a residual analysis in the contingency table was conducted if any significant difference was noted in the test for independence. The internal reliability of question items was assessed with 20 students attending PBL tutorials before the survey.

### Study participants

This study was composed of a qualitative analysis phase with FGDs followed by a quantitative analysis phase with a questionnaire survey. For FGDs, the 4th to 6th year students and junior residents who had completed the PBL program at Mie University Faculty of Medicine were recruited to form a group with four to eight persons. The participants joined the study voluntarily.

A questionnaire survey was conducted to 258 students, 126 5th year and 132 6th year students. A total of 119 out of 258 students responded to the survey (Table 1). The response rate was 46.1%. The female to male ratio was 1:2.5, corresponding with the sex ratio in the whole student body. As for the age composition of respondents, 79.8% were aged 22-25 years, and 20.2% were aged 26 years and above. We further divided the subjects by CBT rank in class. The number of students was similarly distributed as in the whole student body when divided into five groups by every 25 ranks.

**Table 1:** Characteristics of participants in the questionnaire survey

Variable		n (%)
School year		
	5^th^ year	62 (52.1)
	6^th^ year	57 (47.9)
Sex		
	female	34 (28.6)
	male	85 (71.4)
Age		
	22-25 years	95 (79.8)
	26-30 years	16 (13.4)
	31-35 years	5 (4.2)
	36-40 years	2 (1.7)
	41 and above	1 (0.8)
CBT^* ^rank at class		
	1-25	28 (23.5)
	26-50	20 (16.9)
	51-75	23 (19.3)
	76-100	23 (19.3)
	101 and above	24 (20.2)
	missing	1 (0.8)
Total		119 (100)

CBT*, a Computer-based test conducted just after the completion of the PBL program. The test was operated by the Common Achievement Tests Organization in Japan.

This study was approved by the Institutional Review Board at Mie University Faculty of Medicine. The study plan was open to the public on the homepage of Mie University. In the qualitative study with FGDs, informed consent was obtained from the participants before group discussions. The documents applied to the qualitative study were completely anonymized. In the questionnaire survey, an informed consent form was provided to students together with a questionnaire. The answered questionnaire was returned to the investigator if they agreed to participate. The questionnaire survey was anonymous.

### Statistics

The questionnaire constructed in this study was validated for internal reliability with Cronbach’s alpha. An item-total correlation was analyzed with Spearman’s correlation coefficient between the total scores of responses to all questions in each category and all categories. The data in a contingency table were statistically tested for independence with two-sided chi-square test, and then examined with a residual analysis if any significant difference was noted in the test for independence. Significance in the residual analysis was indicated with a p-value for the adjusted standardized residual (ASR).^17^ A p-value less than .05 was regarded as significant where a p-value was estimated from the standard normal distribution. A total score of responses to all questions in each category was calculated and indicated as a percentage to the maximum score (the number of questions x5). Statistical analyses were carried out using IBM-SPSS Statistics 24.0 (IBM Corp., Armark, NY, USA) and BellCurve for Excel (Social Survey Research Information Co. Ltd., Tokyo, Japan).

## Results

### Qualitative study

Four groups were organized with a total of 27 students and residents. Eight medical students in the 4th year (male/female = 4/4), eight (male/female = 4/4) in the 5th year, seven in the 6th year (male/female = 4/3), and four junior residents (male/female = 3/1) working at Mie University Hospital participated in the FGDs. The four groups in the FGDs were organized according to the academic year when the participants joined the PBL program. The textual documents were analyzed with an inductive thematic analysis for each research question at the first phase. We derived three themes from 17 codes in FGDs for the outcome of PBL: effective learning, cognitive thinking, and social skills (Table 2ََA). Discussions on the application of PBL outcome identified three themes from 12 codes: social interaction, continuing learning, and clinical practice (Table 2B). Discussions on challenges of PBL identified five themes from 28 codes: learning resources, assessment, student’s learning behaviors, limitations of application, and reflection of student perspectives (Table 2C). Moreover, discussions on suggestions for better PBL derived three themes from 17 codes: quality of tutors, quality of case-scenarios, and innovation of learning strategy (Table 2D).

Along with the concept of an exploratory sequential mixed method, the phrases specific to each category in the qualitative analysis and the results of the quantitative analysis were indicated as “a joint display” in Table 3. Students acknowledged that the PBL program was effective for acquiring skills of communication, presentation, collaborative learning, cognitive thinking, and clinical reasoning. They also regarded the PBL program as a better learning method than lectures. The following comment was a good example: “Ability of summarizing my idea and speaking precisely would not be improved by learning at lectures while that was improved at PBL tutorials. I believe that such ability is useful after becoming a doctor.” However, some students had hesitation in stating their own opinion before group members and experienced difficulty getting along with other students at PBL tutorials. The following comments showed one student’s thoughts: “I always worried about saying something wrong about my learning achievements.” “I thought about how to get along well with others every time at tutorials.” Accordingly, some students seemed to attach a certain value to lectures. The following comments were given at FGDs: “At Mie University, the general knowledge is learned in lectures, and the symptomology of individual diseases are learned in PBL tutorials.” “It might be nice to have a preparatory lecture at the beginning of a PBL tutorial. If I received a summary lecture, it could be effective.” Furthermore, one student gave the following comment: “PBL works well in Canada, but we need to modify it for Japanese students”, which suggests that the student felt a barrier to effective PBL among Japanese medical students. In the discussions on challenges of PBL and suggestions for PBL, quality of tutoring, case scenarios, group discussions, and assessments were recognized as issues to be improved. Student’s learning behavior was also discussed. The participants of the FGDs commented that there were problems in active participation in PBL tutorials and proper usage of learning resources.

In general, the residents gave more supportive comments for PBL while the students presented more critical opinions. Especially, the 4th year students joining the PBL program at the time of FGDs tended to prefer a lighter workload than a better learning outcome. In this regard, a student commented that “the purpose of PBL was not clearly indicated to students”. In addition, the learning effects of PBL for clinical practice were likely to be recognized after they had become physicians.

### Questionnaire survey

We developed four research questions in the second phase based on the results of the first phase. These questions consisted of 1) learning effects of PBL, 2) social interaction during and after PBL, 3) application of PBL experiences to clinical clerkship, and 4) satisfaction with PBL. Then we generated 24 questions for the questionnaire survey and categorized these questions into four categories according to the research questions in the second phase (Appendix). Before the survey, the internal reliability of 24 questionnaire items was validated among 20 students. The value of Cronbach’s alpha was .875, indicating a good reliability index. The value was also assessed with the 119 students that responded to the questionnaire survey. The value of Cronbach’s alpha was .932. An analysis of the item-total correlation resulted in statistically significant correlations for all of four categories, learning effects of PBL (r = .836, n = 112, p < .001), social interaction during and after PBL (r = .882, n = 112, p < .001), application of PBL experiences to clinical clerkship (r = .868, n = 112, p < .001) and satisfaction of PBL (r = .674, n = 112, p < .001) (Table 3).

**Table 2A:** Results of a thematic analysis - Outcome of PBL

Code	Subtheme	Theme
Approaching learning resources	Knowledge acquisition	Effective learning
Acquiring knowledge		
Learning by listening		
Self-directed learning	Self-learning	
Learning based on self-assessment		
Learning through feedback		
Discussion skills	Skill building	
Questioning and summarizing skills		
Brainstorming leading focus learning	Critical thinking	Cognitive thinking
Acquiring thinking for differentials of the diseases		
Improvement of clinical report writing	Clinical reasoning	
Steps for clinical reasoning		
Evidence-based discussion		
Increase in communication opportunity	Acquiring communication skills	Social skills
Making communication better		
Taking responsibility	Improving social skills	
Expanding networking		

**Table 2B:** Results of a thematic analysis - Application of PBL outcome

Code	Subtheme	Theme
Sympathy to patients	Sympathy	Social interaction
Understanding clinician's roles		
Better communication	Communication	
Motivation to study	Learning behavior	Continuing learning
Spending more time to study		
Self-assessment		
Making proper differential diagnosis	Clinical knowledge & skills	Clinical practice
Application of medical knowledge		
Patient-centered care		
Evidence-based discussion		
Reporting skill		
Skills for producing portfolios		

**Table 2C:** Results of a thematic analysis - Challenges of PBL

Code	Subtheme	Theme
Tutor allocation procedure	Tutoring inconsistency	Learning resources
Tutors' facilitation quality		
Difference between expert and non-expert tutors		
Response to call for support	Less support to student's needs	
Scenarios used in the past	Ineffective scenarios	
Easy scenarios		
Too specific or too broad contents		
Participation of simulated patient	Unfunctional structure of PBL tutorials	
Discussion progress		
Time for self-learning and tutorials		
Self-assessment	Invalid assessments	Assessments
Peer assessment		
Tutor assessment		
Free-riders in group discussion	Unfavorable attitude of peers	Student’s learning behaviors
Selfish group members		
Allocation of group members		
Personality difference		
Knowledge gap among students		
Cultural gap		
Same referencing	Inefficient learning process	
Prior learning condition		
Confusing study objectives		
Time to cope the scenarios provided		
Knowledge gap between the PBL program and clinical clerkship	Limited application for practice	Limitations of the application
Difficulty in the application of learning outcome in PBL		
Regret about PBL	Negative perspectives for PBL	Reflection of student perspectives
Insisting on the PBL program		
Complementary to lectures		

**Table 2D:** Results of a thematic analysis - Suggestion for PBL

Code	Subtheme	Theme
Proper Tutoring	Better tutoring	Quality of tutors
Effective feedback to tutors		
Consistent tutors' facilitation		
Expert tutors' tutoring		
Well-planned tutor allocation		
Training of trainer		
More case scenarios	Better scenarios	Quality of case-scenarios
Scenarios with untypical symptoms		
Upgrading scenarios		
Providing more laboratory data		
Well-planned time scheduling	Rearrangement of PBL tutorials	Innovation of learning strategy
Proper assigning learning topics to members		
Proper and accurate assessments		
Better understanding of study objectives		
Introduction of simulated patients to PBL tutorials	New learning methods	
Case simulation in more active ways		
Teaching aids such as e-learning		

The results of the questionnaire survey are summarized in Table 4. The answers to 24 questions were classified to “strongly agree/agree”, “neutral”, or “strongly disagree/disagree”. A chi-square test of independence found the significant difference in the contingency table (χ^2^ (48, 2848) = 398.3, p < .001). The ASR in the residual analysis for each category of the responses to each question was calculated. A significantly higher ASR for the response of “strongly agree/agree” was found in four out of six questions of Category I, learning effects of PBL (Q1, ASR = 5.901, n = 119, p < .001; Q2, ASR = 2.846, n = 118, p = .004; Q4, ASR = 3.303, n = 119, p < .001; Q6, ASR = 3.591, n = 118, p < .001) and three out of seven questions of Category IV, satisfaction with PBL (Q20, ASR = 4.231, n = 119, p < .001; Q21, ASR = 2.190, n = 119, p = .029; Q22, ASR = 4.046, n = 119, p < .001). A significantly lower ASR for the response of “strongly agree/agree” was found in five out of six questions of Category II, social interaction during and after PBL (Q7, ASR = -3.303, n = 118, p < .001; Q8, ASR = -2.078, n = 119, p = .038; Q9, ASR = -3.006, n = 119, p = .003; Q10, ASR = -2.449, n = 119, p = .014; Q11, ASR = -5.790, n = 119, p < .001). Among five questions of Category III, application of PBL experiences to clinical clerkship, two had a significantly higher ASR for the response of “strongly agree/agree” (Q14, ASR = 4.973, n = 119, p < .001; Q17, ASR = 4.417, n = 119, p < .001) and another two had a significantly lower ASR (Q13, ASR = -4.657, n = 116, p < .001; Q16, ASR = -5.975, n = 119, p < .001).

Table 3 shows a joint display of the results from qualitative and quantitative analyses in an exploratory sequential method. The percentage of total scores to the maximum value in each category is indicated. In accordance with the results of the residual analysis for all questions, Categories I (learning effects of PBL) and IV (satisfaction with PBL) showed higher values (68.9 and 67.9%) and Category II (social interaction during and after PBL) had the lowest value (57.7%). These findings mostly reflected the results of the qualitative study; however, opposite views were observed in the qualitative study.

## Discussion

In our curriculum, students in the preclinical years attend the three different types of classes: PBL tutorials, TBL, and large lecture classes. In this study, we aimed to clarify the students’ perception of PBL in this situation using an exploratory sequential mixed method. Furthermore, we aimed to elucidate the challenges of PBL for Japanese medical students.

Students appreciated that PBL was an effective learning strategy. A positive response was significantly higher for the question on self-learning skills and clinical reasoning skills. These findings are consistent with previous studies.^18^^-^^22^

**Table 3:** A joint display of the results from the exploratory sequential mixed method

Qualitative study phase				Quantitative study phase		
Discussion topics at FGD	Themes identified by the thematic analysis	Specific phrases at FGDs for each theme		Category the question number	% total score in each category*	Item-total correlation†
Outcome of PBL	Effective learning	- Ability of summarizing my idea and speaking precisely would not be improved by learning from a lecture; however, that was improved at PBL. I believe that such an ability is useful after becoming a doctor.		Learning effects of PBL Q1-6	68.9% (2445/3550) n = 119	r=.836 n=112 p<.001
		- I could learn beyond my self-learning at each session of the group discussions. It seems to be a better way to learn huge volumes of medical knowledge.				
		- At Mie University, the general knowledge is learned in lectures, and the symptomology of individual diseases are learned in PBL tutorials.				
		- The discussion sometimes kept on going even if I did not understand the discussion.				
	Cognitive thinking	- PBL-tutorials made me understand how the knowledge of basic medical science is applied to clinical practice. We did not know the importance of basic medical science and knowledge so much for clinical practice.				
		- At a PBL-tutorial, the sequence goes in a reverse direction of the contents of textbooks. It is same as the diagnosis of real patients. I could learn a way of thinking which is needed in a clinical setting.				
Application of PBL outcome	Social skills	- I got to feel that it was easy to have a discussion with my classmates even if my opinion might be wrong through the PBL program.		Social interaction during and after PBL Q7-12	57.7% (2047/3550) n = 119	r = .882 n = 112 p < .001
		- I gained the ability to explain after considering other’s level of understanding.				
		- I always worried about saying something wrong about my learning achievements. Every time I honestly hoped that the tutorial would be finished as soon as possible.				
	Social interaction	- I thought about how to get along well with others every time at a tutorial. I think such ability was trained at tutorials, but I really disliked it.				
		- The good atmosphere motivates students to communicate freely. If a few members talk too much, the group may not work well.				
	Continuing learning	- I became to summarize clinical information effectively. The PBL program was a good pre-clinical training.				
	Clinical practice	- If I join clinical rotations without such learning, I may have a lot of trouble at a hospital.		Application of PBL experiences to clinical clerkship Q13-17	63.4% (1886/2975) n = 119	r = .868 n = 112 p < .001
Challenges of PBL / Suggestion for PBL	Learning resources	- Since many students use the same textbook, the contents of group work overlapped. There is no meaning to discussing together.		Satisfaction with PBL Q18-24*	67.9% (2826/4165) n = 119	r = .674 n = 112 p < .001
		- I learned about searching PubMed and looked for more sources. Because I had read only textbooks, the source of information expanded.				
		- It is hard to understand physical examinations on paper. I recommend that more visual materials should be provided.				
	Assessments	- Too many assessment items disturb student’s proper assessment.				
		- Peer assessment tends to be done with bias by the preconception of each student.				
	Student’s learning behaviors	- I made a copy of an easy text as part of my portfolio. Everyone is copying the contents.				
		- Students who benefit most from group learning are those who do not study by themselves before group work.				
		- Since we are passive, there is little feedback from the tutor.				
	Quality of tutors	- Some tutors are not specialized physicians. In such cases, the tutor was less involved in the activities. I hoped the tutor would be more active.				
		- If tutors improve their facilitation skills, PBL would be completely different. Tutor training should be required.				
		- Some tutors are very enthusiastic. Such tutors understand the scenario and know the teaching points properly. But some may not well understand the scenario. The standardization of tutors’ teaching skills may be challenge for better PBL tutorials.				
	Quality of case-scenarios	- Since the same scenarios are used every year, some students get the information from senior students. They deal with tutorials without learning anything.				
	Innovation of learning strategy	- Someone should play the role of the patient indicated in the scenario. A tutor can make a good reaction like an actual patient.				
		- It might be nice to have a preparatory lecture at the beginning of the PBL tutorials. If I received a summary lecture, it could be effective.				
		- We should put more emphasis on patient's complaints than laboratory data.				
	Reflection of student perspectives	- The purpose of PBL was not clearly indicated to students.				
		- PBL works well in Canada, but we need to modify it for Japanese students. The condition of PBL tutorials should be changed so that we can talk comfortably.				
	Limitations of the application	- There was a gap in knowledge between learning at PBL and clinical practice.				
		- We should learn more about the process of patient care.				

*A total score of responses to all questions in each category was counted and expressed as % total score of the maximum value. Deficit values at each question were 1 in Q2, 1 in Q3, 1 in Q5, 1 in Q6, 1 in Q7, and 3 in Q12. †An item-total correlation was analyzed between the total scores of responses to all questions in each category and all categories. The results were expressed as a correlation coefficient and p-value. The score at Q24 was reversely counted in data analysis as it was a negative question.

However, the positive response was not significantly higher for the question on critical thinking ability. This may be related to the learning attitude of the students as discussed later. Pre-clinical education is expected to foster the abilities needed for clinical clerkship. But this study showed that there were arguments for and against on the application of PBL experiences to clinical clerkship among students. Significantly more students disagreed with the question “PBL tutorials were instructive for me to improve my study-style”. Most interestingly, social interaction in PBL was not perceived affirmatively as there were significantly fewer students who agreed that PBL improved communication skills, responsibility and accountability at group discussion, and social networking. Moreover, the qualitative study indicated the passive attitude to group work and preference for the traditional style of learning in some students. A previous study from a Japanese medical school reported that the majority of students were satisfied with PBL; however, 5% preferred traditional lecture-style learning.^23^ In Japan, students may not have had exposure to learning through open discussion, debating, or group work since childhood. Moreover, they are also not familiar with constructive criticism. Such a learning culture may influence Japanese students’ attitude for learning. To improve this situation, we should emphasize the importance of active learning in medical education and provide some introductory sessions before the PBL program.

Previous studies reported that PBL achieved better learning outcomes in various aspects.^4^^-^^6^ However, the findings in the present study were not completely consistent with these previous studies. The passive attitude of students may be associated with these negative findings. The qualitative study extracted other problems in the quality of tutoring, case scenarios, and assessments. Davis et al. reported that in small-group teaching sessions, student groups facilitated by content-experts had higher levels of satisfaction and higher examination scores than those led by non-experts.^24^ The selection of tutors is one of the critical components of PBL; however, content-experts are not always available due to the limited resources of teaching staff. We should enhance the tutoring ability of teachers effectively through faculty development. For better case scenarios, the participants of FGDs suggested the use of visual materials and simulated patients. The production of case scenarios that are attractive to students is critical for effective PBL. An effective assessment is also important to facilitate student’s learning. The results of FGDs suggested that students looked for a fair assessment and timely feedback by tutors. The assessment system should be continuously improved with feedback from students; however, previous studies reported that the majority of students were not satisfied with the evaluation system in medical education curriculum.^25^^,^^26^

The findings in the present study need to be considered in the context of its limitations. FGDs were conducted by students and residents participating voluntarily but not randomly assigned. Thus, this is likely to have resulted in bias towards choosing more active students.

**Table 4:** Results of the residual analysis for questions in the questionnaire survey

Category	Question No.	Strongly agree/Agree		Neutral		Disagree/Strongly disagree		Total number of responses
		n (%) Expected value ASR*	p-value	n (%) Expected value ASR*	p-value	n (%) Expected value ASR*	p-value	
Learning effects of PBL	Q1	88 (73.9)	< .001	24 (20.2)	.039	7 (5.9)	< .001	119
		56.20		34.10		28.71		
		5.901		-2.069		-4.700		
	Q2	71 (60.2)	.004	28 (23.7)	.232	19 (16.1)	.040	118
		55.73		33.81		28.46		
		2.846		-1.195		-2.058		
	Q3	31 (26.3)	< .001	38 (32.2)	.389	49 (41.5)	< .001	118
		55.73		33.81		28.46		
		-4.608		0.862		4.465		
	Q4	74 (62.2)	< .001	31 (26.1)	.526	14 (11.8)	.002	119
		56.20		34.10		28.71		
		3.303		-0.634		-3.184		
	Q5	60 (50.8)	.421	36 (30.5)	.649	22 (18.6)	.155	118
		55.73		33.81		28.46		
		0.796		0.451		-1.406		
	Q6	75 (63.6)	< .001	29 (24.6)	.322	14 (11.9)	.002	118
		55.73		33.81		28.46		
		3.591		-0.989		-3.145		
Social interaction during and after PBL	Q7	38 (32.2)	< .001	32 (27.1)	.710	48 (40.7)	< .001	118
		55.73		33.81		28.46		
		-3.303		-0.372		4.248		
	Q8	45 (37.8)	.038	35 (29.4)	.853	39 (32.8)	.026	119
		56.20		34.10		28.71		
		-2.078		0.185		2.229		
	Q9	40 (33.6)	.003	31 (26.1)	.526	48 (40.3)	< .001	119
		56.20		34.10		28.71		
		-3.006		-0.634		4.178		
	Q10	43 (36.1)	.014	44 (37.0)	.042	32 (26.9)	.476	119
		56.20		34.10		28.71		
		-2.449		2.029		0.713		
	Q11	25 (21.0)	< .001	39 (32.8)	.315	55 (46.2)	< .001	119
		56.20		34.10		28.71		
		-5.790		1.005		5.694		
	Q12	56 (47.1)	.971	31 (26.1)	.526	32 (26.9)	.476	119
		56.20		34.10		28.71		
		-0.037		-0.634		0.713		
Application of PBL experiences to clinical clerkship	Q13	30 (25.9)	< .001	33 (28.4)	.961	53 (45.7)	< .001	116
		54.78		33.24		27.98		
		-4.657		-0.049		5.486		
	Q14	83 (69.7)	< .001	21 (17.6)	.007	15 (12.6)	.003	119
		56.20		34.10		28.71		
		4.973		-2.683		-2.968		
	Q15	47 (39.5)	.088	38 (31.9)	.424	34 (28.6)	.252	119
		56.20		34.10		28.71		
		-1.707		0.800		1.147		
	Q16	24 (20.2)	< .001	49 (41.2)	.002	46 (38.7)	< .001	119
		56.20		34.10		28.71		
		-5.975		3.054		3.745		
	Q17	80 (67.2)	< .001	23 (19.3)	.023	16 (13.4)	.006	119
		56.20		34.10		28.71		
		4.417		-2.273		-2.751		
Satisfaction with PBL	Q18	37 (31.1)	< .001	35 (29.4)	.853	47 (39.5)	< .001	119
		56.20		34.10		28.71		
		-3.563		0.185		3.962		
	Q19	52 (43.7)	.436	44 (37.0)	.042	23 (19.3)	.217	119
		56.20		34.10		28.71		
		-0.779		2.029		-1.235		
	Q20	79 (66.4)	< .001	29 (24.4)	.297	11 (9.2)	< .001	119
		56.20		34.10		28.71		
		4.231		-1.044		-3.834		
	Q21	68 (57.1)	.029	38 (31.9)	.424	13 (10.9)	< .001	119
		56.20		34.10		28.71		
		2.190		0.800		-3.401		
	Q22	78 (65.5)	< .001	37 (31.1)	.552	4 (3.4)	< .001	119
		56.20		34.10		28.71		
		4.046		0.595		-5.350		
	Q23	58 (48.7)	.738	37 (31.1)	.552	24 (20.2)	.308	119
		56.20		34.10		28.71		
		0.334		0.595		-1.019		
	Q24**	63 (52.9)	.207	34 (28.6)	.984	22 (18.5)	.147	119
		56.20		34.10		28.71		
		1.262		-0.020		-1.452		

*ASR, adjusted standardized residual

**Q24 was a negative question.

The independence in the contingency table was significant. Chi-square test, χ^2^ (48, 2848) = 398.3, p < .001.

The adjusted standardized residual with p < .05 was regarded as statistically significant in the residual analysis.

The respondents in the questionnaire survey may also have such characteristics. The collection rate at the survey was relatively low; however, the ratio of respondents by school year, sex, or age composition reflected the ratios of the whole student body. In addition, the CBT rank divided by every 20% matched the actual distribution. Thus, we assumed that the participants represented the student cohort planned in the study design. The present study was a cross-sectional study; therefore, we could not assess the change of students’ perception longitudinally with the progress of their experiences in clinical practice.

For the qualitative study, the results could be influenced by the investigators’ values and beliefs although we tried to avoid subjective decisions. Furthermore, as the medical education curriculum varies from school to school, the findings in this study may not be applicable to other schools.

## Conclusion

PBL was perceived as an effective learning strategy in the pre-clinical curriculum of a Japanese medical school. However, some medical students had problems with social interaction, which made it difficult for them to actively participate in group work. Moreover, some students preferred lectures even if the passive learning approach produced less learning effects. Although students were generally satisfied with PBL, the qualitative study found dissatisfaction with the quality of tutoring, case scenarios, and assessments.

### Acknowledgements

The study was supported by an official budget from Mie University Graduate School of Medicine. The fund covered all costs related to the study.

### Conflict of Interest

The authors declare that they have no conflict of interest.

## Supplementary material

Supplementary file 1 Appendix. Questions in the questionnaire constructed according to the results of the qualitative analysis
