# Supplementary material for: Students’ perception of problem-based learning at a Japanese medical school: an exploratory sequential mixed method
Source: Int J Med Educ. 2022 Dec 26;13:322–34. doi: 10.5116/ijme.6399.dee1 (PMC9911279; doi:10.5116/ijme.6399.dee1)
Supplement: Supplementary file 1 — Appendix. Questions in the questionnaire constructed according to the results of the qualitative analysis [file ijme-13-322-S1.pdf]

Appendix

Questions in the questionnaire constructed according to the results of the qualitative analysis

Category I	Learning effects of PBL
Q1	Did PBL tutorials help you to acquire medical knowledge?
Q2	Did your self-learning skills improve through PBL tutorials?
Q3	Were PBL tutorials instructive for you to improve your study-style?
Q4	Did you appreciate PBL tutorials as an important learning method?
Q5	Did PBL tutorials help you improve critical thinking ability?
Q6	Did PBL tutorials help you improve clinical reasoning skills?
Category II	Social interaction during and after PBL
Q7	Did your communication skills improve during and after studying in PBL tutorials?
Q8	Was your responsibility/accountability at group discussions enhanced through PBL tutorials?
Q9	Was your social networking status enhanced by PBL tutorials?
Q10	Did you become able to sympathize with patients?
Q11	Did you become able to understand more about social aspects of clinical practice and roles of clinicians?
Q12	Did you become better able to communicate with people at hospital?
Category III	Application of PBL experiences to clinical clerkship
Q13	Can you make clinical reasoning efficiently?
Q14	Do you spend more time for self-learning with motivation in clinical rotations?
Q15	Are you aware of a patient-centered approach?
Q16	Did you learn clinical medicine effectively prior to hospital training by simulating clinical practice?
Q17	Can you use the knowledge that you earned at PBL for clinical training without any difficulty?
Category IV	Satisfaction with PBL
Q18	Were you satisfied with tutoring of tutors in PBL tutorials?
Q19	Were you satisfied with PBL case-scenarios in PBL tutorials?
Q20	Were you satisfied with student's peer assessment in PBL tutorials?
Q21	Were you satisfied with tutors' assessment in PBL tutorials?
Q22	Were you satisfied with group discussions at tutor time?
Q23	Did you enjoy the participation in learning in PBL tutorials?
Q24	Did PBL program have little or no involvement of student perspectives? *

Twenty-four questions were classified into four categories according to the results of the qualitative analysis
*, The score was reversely counted in data analysis because it was a negative question.
